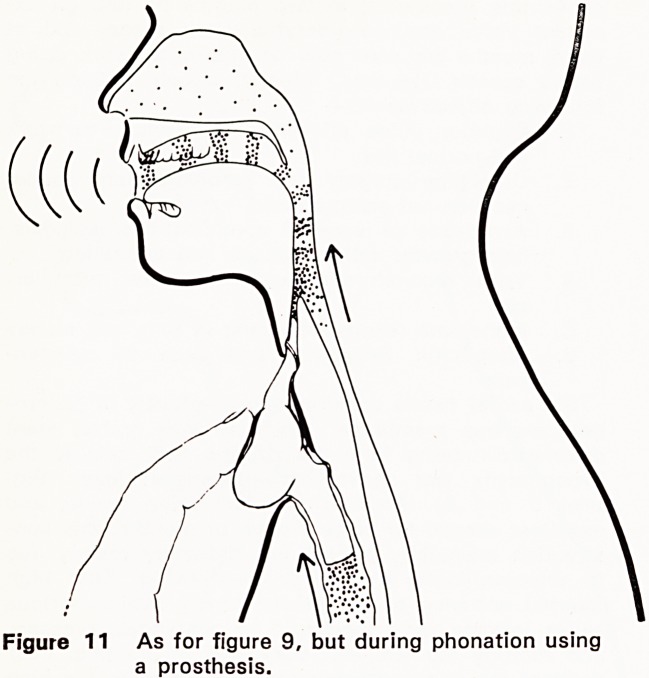# New Voices for Old

**Published:** 1975-01

**Authors:** Nigel Edwards

**Affiliations:** Consultant Ear, Nose and Throat Surgeon, Frenchay and Southmead Hospitals


### New Voices for Old

### Restoration of effective speech after Laryngectomy by the Pulmonary Air Shunt-Vocal Fistula Principle

Nigel Edwards, M.A., M.B., B.Chir., F.R.C.S.

Consultant Ear, Nose and Throat Surgeon,
Frenchay and Southmead Hospitals

(based on a presentation at the Postgraduate Clinical Meeting, Southmead Hospital, July 26th, 1974)

Total laryngectomy is still an important and effective method of controlling laryngeal cancer which is locally very advanced, or where radiotherapy has already failed. Where the tumour is endo-laryngeal, a relatively favourable situation, a cure rate of at least 50% can be expected, and nearly all cases will have a fairly long survival in otherwise reasonable health. Where laryngectomy is part of the surgical treatment of hypopharyngeal cancer, however, the prognosis for cure remains poor, although useful palliation is often achieved; life expectancy is generally rather short and problems in achieving vocal rehabilitation are formidable. It is estimated that every year in England and Wales about 300 laryngectomies are carried out for cancer. In the U.S.A., where there is a greater bias towards radical surgery, about 2,000 laryngectomies are done yearly.

The operation, first carried out successfully a century ago in Vienna by Billroth, carried a high operative mortality in the early days, but today is a routine surgical procedure with very low mortality and complication rate. For the patient, however, it is a crippling mutilation and devastating experience which will inevitably produce a profound alteration in the quality of his life. The ability to communicate verbally is a most precious human asset. Clearly the prime need in rehabilitation after laryngectomy is the early restoration of adequate speech.

Since the turn of the century **oesophageal speech** has been almost universally accepted as the best available method of post-laryngectomy speech rehabilitation, with electronic or mechanical vibratory devices ("electrolarynx") coming far behind. The electrolarynx is generally offered only to those who fail to learn oesophageal speech. The voice produced is non-human, and "robot-like". In the post-war period, increasing dissatisfaction with oesophageal speech, together with remarkable technical advances in reconstructive surgery made possible by the control of infection and by modern anaesthesia, have stimulated efforts to produce a more effective voice by surgical reconstruction after removal of the larynx. Today these efforts are beginning to bear fruit. This paper reviews some promising recent surgical advances, and refers to the author's own work.

### Laryngectomy and the Vocal Mechanism

In order to understand the principle of these new methods, let us first consider in simplified form the essential effector components of the intact vocal mechanism and how removal of the larynx affects this system.

The basic mechanisms for speech production are:

1) Generator (normally lungs and respiratory muscles)
2) Vibrator (normally vocal cords/glottis)
3) Vocal tract (with its modulators and articulators)

They are disrupted by total laryngectomy because the operation:—

1) Disconnects the generator
2) Removes the normal vibrator
3) Leaves the vocal tract functionally intact

In normal laryngeal speech, the energy of expired air sets the apposed vocal cords into vibration which is passed on to the air column of the vocal tract as sound waves of definite fundamental frequency and harmonic pattern. The basically feeble laryngeal sound waves are selectively amplified, modified and articulated in the vocal tract into what we recognise as highly individual speech. The vocal tract is the only indispensable member of the trio, and equally can modify sound vibrations of non-laryngeal origin.

Phylogenetically, the larynx is primarily a sphincter mechanism to protect the lungs from aspiration of food and secretions (Negus, 1949); its phonatory function, so subtle in man, is a secondary development. After standard total laryngectomy, an external tracheostomy is customarily established to protect the lungs and ensure a free airway (Figure 1). This anatomical separation of food and air passages, so necessary in earlier times, led inevitably to the development of oesphageal speech (Figure 2), relatively small amounts of air being swallowed and eructated in rapid alternation. The cricopharyngeal constriction usually forms the substitute glottis; the resulting sound vibrations are processed into speech by the intact vocal tract mechanism.

**Figure 1** Anatomy after standard total laryngectomy, and oesophageal speech—intake of air.

**Figure 2** Oesophageal speech—air expulsion and speech.

**Oesophageal Speech**

This highly unphysiological principle has restored passable (and sometimes excellent) speech to many thousands of laryngectomees over many years. It is universally applicable, and requires no special reconstructive surgery, and no apparatus. Its effectiveness has been enhanced by the growth of "Laryngectomee Clubs", whose members help and encourage each other. The U.S.A. have been particularly active in establishing such clubs, as have the International Association of Laryngectomees to which their own clubs, and most others are affiliated. Bristol has its own active "Missing Chords Club" which is under the energetic direction of Sister Millicent Painton, M.B.E., and meets regularly in Bedminster.

**Oesophageal Speech** however, has some serious inherent disadvantages, consequent upon disconnection of the physiological generator, expired pulmonary air; and the method would not seem capable of further improvement. The disadvantages are:—

1) Unphysiological—speech and respiration dissociated.
2) New technique to be learned, often slowly.
3) Power of voice is limited.
4) Interference noise from air swallowing and respiration.
5) Considerable failure rate.

The voice produced is thus far from natural in its low pitch, harshness and lack of normal fluency and power. For most who succeed, months of hard efforts are usually required. But most serious of all is the disturbingly high failure rate—between 25 per cent and 33 per cent fail to achieve an adequate standard enabling them to converse intelligibly with strangers. Females, the elderly, and cases of pharyngolaryngectomy requiring repair by stomach, colon, or skin, are particularly liable to fail with the method.

**Alaryngeal Speech: Other Methods**

**A summary of the methods available** after total laryngectomy for introducing suitable sound vibrations into the vocal tract for speech is as follows:—

I. Electronic or Mechanical Vibrators
   - Internal
   - External through neck tissues
   - External through tube in fistula or mouth

II. Introduced Air
   - Swallowed and returned ("oesophageal speech")
   - Expired air via internal fistula (e.g. Asai)
   - Expired air via external fistula and connecting tubes

The two groups are fundamentally different.

**Of the electronic—mechanical vibrator devices,** the electrolarynx (Figure 3), which is held against the neck skin over the pharynx, is most frequently used. The Tait Oral vibrator is attached to a dental plate but wires connecting it to its power source pass out through the mouth. Efforts have been made, with some success, to introduce electronic vibrations directly into the pharyngeal lumen through a narrow pharyngocutaneous fistula. All these devices produce a monotonous "mechanical" voice and control generally has to be manual—their lack of popularity is not hard to understand.

**Figure 3** Electrolarynx ("Bart's Vibrator").

**Figure 4** Accidental tracheo-pharyngeal fistula used for fistula speech.

**Air is the motive force in the second group.** The deficiencies of oesophageal speech due to disconnection of the pulmonary air have already been considered. Reconnection of the pulmonary air for speech purposes immediately produces remarkable results; the presence of a recognisable specific vibratory structure, surprisingly, does not seem to be essential for phonation: air turbulence readily occurs at many sites in the vocal tract producing sound vibrations.

**Historical Survey**

The effects of pulmonary air were recognised 50 years ago, when a patient with an accidental unwanted tracheopharyngeal fistula (dangerous from the risk of pulmonary aspiration) blew expired air through his fistula, producing immediate, clear and fluent speech (figure 4).

Little was done to exploit this principle, apart from some isolated, crude and unscientific pre-war attempts, until the nineteen fifties. Briani (1958) of Verona, Italy, was the first to make a deliberate pharyngocutaneous fistula and fit a prosthetic connecting apparatus for manual operation of a tracheo-pharyngeal air shunt which produced fistula speech of good quality and fluency (Figures 5 and 6). A few years later, Conley, 1969, in New York, using some ingenious surgical tracheo-oesophageal fistulas constructed from skin, local mucous membrane, or free saphenous vein grafts, designed with valvular properties, confirmed Briani's reports of immediate restoration of voice superior in quality, power and fluency to oesophageal speech. He also, however, honestly reported the formidable problems attending construction of vocal fistulas of this type—notably the difficulties of reliable surgical construction in an area damaged by radical cancer surgery and often irradiated; the liability of fistulas to leak saliva, liquids and food; the tendency of such fistula tracks to fibrous stenosis and occlusion, or less commonly to atrophy and dilatation; and the problems of controlling the phonatory air-shunt (manual control being rightly considered undesirable and unacceptable).

In the **mid-nineteen sixties,** further impetus was given by Asai (1972) of Japan with a three-stage vocal fistula construction, using a long buried skin tube entering high into the pharynx (Figure 7). Manual control of the air shunt is needed. I have heard excellent speech from this construction but serious disadvantages are the multiple-stage reconstructive surgery, saliva leakage, the difficulties of maintaining patency of the track, and manual control of the air shunt. There have been many imitators and modifiers of the Asai Vocal Reconstruction, but the basic problems remain.

**More recently,** the **relative advantages of an open or external type of vocal fistula** (as opposed to Asai's closed or internal tracheo-pharyngeal fistula) have been appreciated (Figures 5, 6, 8, 9, 10, 11). External fistulas are in general simpler to construct and may be made at the time of laryngectomy; they are accessible to bouginage, and stenosis or occlusion can be prevented. They still tend to leak saliva, and a valved connecting prosthesis now becomes obligatory: this is cosmetically disadvantageous but can be used to incorporate valves which will operate the phonatory air-shunt automatically or by remote control. Taub in New York already has such a valvular prosthesis on the market (Taub and Bergner, 1972). He constructs

**Figure 5** Low pharyngo-cutaneous vocal fistula.

**Figure 7** Asai vocal fistula (internal, direct type) during phonation

**Figure 6** Low pharyngo-cutaneous fistula with prosthesis during phonation.

**Figure 8** Oesophago-cutaneous vocal fistula (as in Taub's vocal reconstruction).

a skin tube fistula from laterally above the left clavicle into the upper oesophagus below the cricopharyngeal sphincter; this constriction forms the effective vibratory mechanism, as it does in oesophageal speakers using the burping methods (Figs. 1, 2, 8 and 9). Speech by the Taub air bypass method is "oesophageal" in character, but powerful, fluent and readily intelligible. His results are impressive but the surgical construction carries some hazards, with occasionally dangerous complications (Figs. 8 and 9). Sisson and McConnell (unpublished data) of Chicago, and the author (Edwards, 1974, Edwards, 1975) independently, are presently working on the principle of a vocal fistula which runs from the skin surface into the pharynx above the cricopharyngeal sphincter, keeping well clear of the dangerous area at the root of the neck, where the great vessels are a surgical hazard. The voice no longer has the harsh oesophageal quality but is more highly-pitched. Development of an automatic valvular connecting prosthesis is proceeding, to allow as desired quiet respiration, phonation, and elimination of secretions by coughing, without the need for manual control.

**Personal Experiences**

In the past four years I have carried out external vocal fistula constructions on six patients in a pilot study at Frenchay Hospital. Most of these have been secondary, later constructions after the laryngectomy. On Case No. 3, however, a man of 60 whose glottic carcinoma had persisted after radio-therapy, I first carried out a one-stage vocal fistula construction at the time of laryngectomy, using a tube of pharyngeal mucous membrane. Healing was uneventful in spite of the radiation, and at five weeks he spoke clearly when fitted with an early type of silicone rubber connecting prosthesis; at two months he had an excellent voice and considerable pitch range; and at three months he was able to return to work using fistula speech. He thus, initially, largely fulfilled the following objectives :—

1. Superior voice allowing early return to work and normal life
2. One-stage surgery and reconstruction: cancer excision not compromised
3. Avoidance of surgical complications, stenosis, hair growth, saliva leakage and aspiration
4. Vocal reconstruction applicable after radiotherapy
5. Immediate results: low cost in time and money
6. Simplicity: Reliability and ease of maintenance

My earlier fistula constructions (variously of pharyngeal mucous membrane, and free skin grafts) were downward-running from the midline neck skin to the hypopharynx just above crico-pharyngeal level (Figures 5 and 6). After initially gratifying results and excellent speech for some weeks, or months, this construction eventually proved unsatisfactory mainly due to uncontrollable leakage on swallowing. That high internal entrance of the fistula largely avoids serious saliva leakage, was suggested by pharyngeal pressure and motility studies, and confirmed in practice. Four patients are at the present time converted to the **high subglottic fistula** (using a tubed flap of tongue mucosa in one, and free skin grafts in the rest), and leakage is insignificant (Figures 10 and 11). Voice quality is a little less good than with the original low fistula. In all my fistula speakers, the fistula track itself has provided the vibratory substitute glottis. The general voice pitch has been well above that of oesophageal speakers and closer to the normal male voice level.

**Figure 9** As for figure 8, but during phonation using a prosthesis.

**Figure 10** High subglottic external vocal fistula.

**Figure 11** As for figure 9, but during phonation using a prosthesis.

**My experiences with this pilot series,** particularly with Patient No. 3, convinced me that the tracheo-pharyngeal air shunt principle held the promise of offering reliable and rapid restoration of a voice of reasonably natural character to all laryngectomees; the external type of construction with valved prosthesis seemed better able to achieve the aims mentioned, particularly the immediate one-stage surgery which is so especially desirable for those patients whose survival is likely to be short. It has been clear that an intensive research and development programme is needed to design a satisfactory automatically-functioning prosthesis which can be commercially manufactured—the basic design of the author's prototype prosthesis is now under provisional U.K. patent cover—and the optimal type of vocal fistula reconstruction. A two-year programme funded by the National Research Development Corporation, with further assistance from the Van Neste Foundation and a private donor, is arranged to commence in January 1975, to fulfil these immediate objectives. No other project of this kind exists in Britain. Basic research, much of it new, will be carried out at the same time into the little-understood mechanisms of fistula speech: the site and mode of function of the substitute glottis will receive detailed attention; the fundamental frequency and harmonic spectrum of various types of alaryngeal speech will be examined; and the feasibility of incorporating a voluntarily-controlled neuromuscular mechanism into the substistute glottis for wide pitch variability and vocal Inflection will be considered. We cannot expect to reproduce accurately all the subtleties of which the human larynx is capable, but we aim at an approximation. The female laryngeal voice will certainly prove more difficult to imitate than the male as illustrated by some mean fundamental frequencies:

Oesophageal speech (male and female)—63 Herz.
Asai Fistula speech (reported by Snidecor)—average 105 Herz.
Laryngeal Speech (Male)—132 Herz.
Laryngeal Speech (Female)—220 Herz.

---

**"Reconstructive Laryngectomy"—New Developments (Serafini, 1971)**

This review would not be complete without mentioning recent new developments in surgical reconstruction after laryngectomy, which have largely stemmed from the experimental and clinical work of Serafini in Padua, Italy. After removal of the larynx, in selected cases, he reimplants the tracheal stump directly into the pharynx high beneath the hyoid and tongue base. He has succeeded in a small number of cases in using this tracheopharyngeal communication not only for phonation but also for complete respiratory needs, dispensing with a tracheostomy entirely. He relies, for protection of the lungs, on the normal movements of the hyoid appartus and tongue during swallowing. The requirements for respiration on the one hand (a wide "neo-glottis") and for good voice, and avoidance of aspiration (a narrow "neo-glottis") are opposite, and the balance needed is a delicate and critical one.

It is too early yet to evaluate this interesting development which depends upon preservation of functionally intact musculo-skeletal structures. This may often prove incompatible with adequate cancer treatment by radical surgery with or without radiation therapy. It does, however, clearly show the importtance of preserving where possible the vital physiological mechanisms of the upper food and air passages; the loss of one mechanism may be amply compensated by the remaining function of the others.

**Summary of present position**

I believe that these recent developments are leading us into a new era in laryngeal cancer surgery, and with it a new philosophy, in which our aim will not be merely to cure the patient of his cancer but also to restore him swiftly to a normal life of verbal communication with his fellow men. Today, failure to rehabilitate one third of the laryngectomised adequately by oesophageal speech can not be considered acceptable.

At the present time, no entirely satisfactory or universal method of vocal reconstruction is in operation, and difficult problems still have to be solved. The Serafini type of "Reconstructive Laryngectomy" is a bold and imaginative step towards the ideal in rehabilitation, with restoration of both speech and respiration through the normal passages (Serafini, 1971; Arslan, 1972). Its effectiveness and safety have yet to be evaluated in the long term.

My own project has less comprehensive aims with the external vocal fistula and valved prosthesis method which should prove to be simple, reliable, safe and universally applicable, as well as rapidly effective. Reliance on a prosthesis, which by good design can be made inconspicuous and cosmetically acceptable, will, it is hoped, be considered a small price to pay for the immense benefits of fistula speech. Intensive work is now needed to bring the method into regular clinical practice. It is certainly premature to be dogmatic over indications for primary or secondary vocal reconstruction surgery, or the ultimate position of oesophageal speech. For the present all available methods should be used to obtain speech, and the pitiable plight of the speechless larygectomee should soon become a thing of the past.

**Acknowledgements**

My thanks are due to the Medical Illustration Department of Southmead Hospital, and especially to Mr. A. Cottrell for his diagrams. I acknowledge with thanks the research grant given by the National Research Development Corporation and supplemented by the Van Neste Foundation and a private donor; and I thank the Wellcome Trust for their recent Wellcome Travel Grant which enabled me to study the Serafini technique of Reconstructive Laryngectomy in Italy; also Frenchay and Southmead Hospitals for additional travel grants.

**References**

ARSLAN, M.: (1972) "Reconstructive Laryngectomy: report on the first 35 cases", **Annals of Otology, Rhinology, Laryngology, 81**, 479.

ASAI, R. (1972). Laryngoplasty after total laryngectomy. **Archives of Otolaryngology, 95**, 114-119.

BRIANI, A. A.: (1958) "Il ricupero sociale dei laringectomizzati attraverso un metodo personale operatorio" **Medicina Sociale, 8**, 265-269.

CONLEY, J. J. (1969) "Surgical Techniques for the Vocal Rehabilitation of the post-laryngectomised Patient". **Transactions of the American Academy of Ophthalmology and Otology, 73**, 288-299.

EDWARDS, N.: (1974) "Post-laryngectomy Vocal Rehabilitation". **Journal of Laryngology and Otology, 88**, 905-918.

EDWARDS, N.: (1975) "Post-laryngectomy Rehabilitation by the External Fistula Method: Further Experiences". **Laryngoscope, 85**, 690-699.

NEGUS, V. E.: (1949) **"The Comparative anatomy and physiology of the Larynx"**, Hafner Publishing Co., New York and London (reprinted 1962).

SERAFINI, I.: (1971) "A case of total laryngectomy with maintained natural breathing operated with a personal technique". **Panminerva Medica, 13**, 377-386.

SNIDECOR, J. C.: et al. **Speech Restoration of the Laryngectomised**, 2nd edition, 1968 Chas. C. Thomas, Springfield, Illinois.

TAUB, S. and BERGNER, L. H.: (1972) "Air Bypass Voice Prosthesis for Vocal Rehabilitation of Laryngectomes". **American Journal of Surgery, 125**, 748. 748.

## Figures and Tables

**Figure 1 f1:**
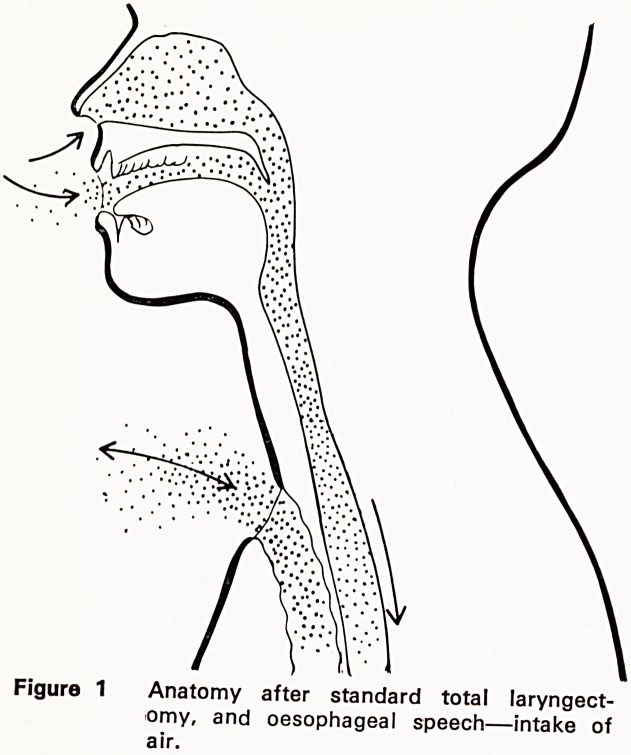


**Figure 2 f2:**
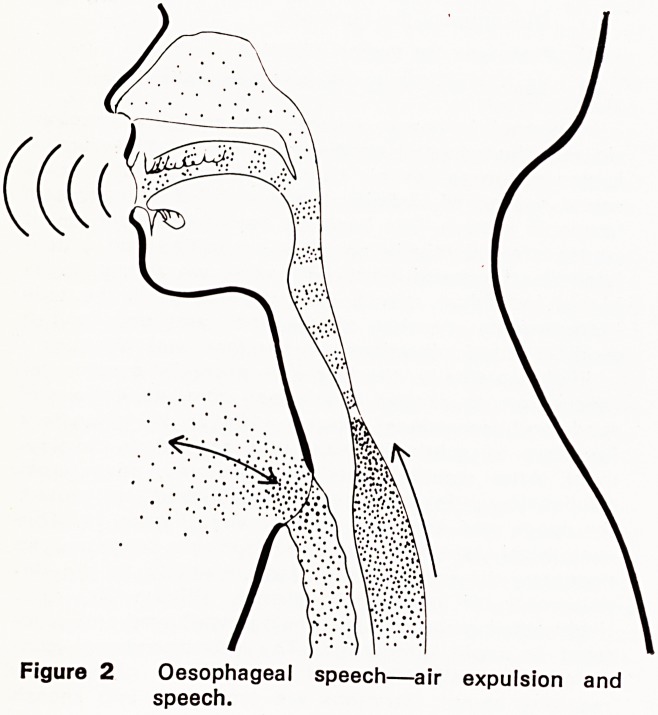


**Figure 3 f3:**
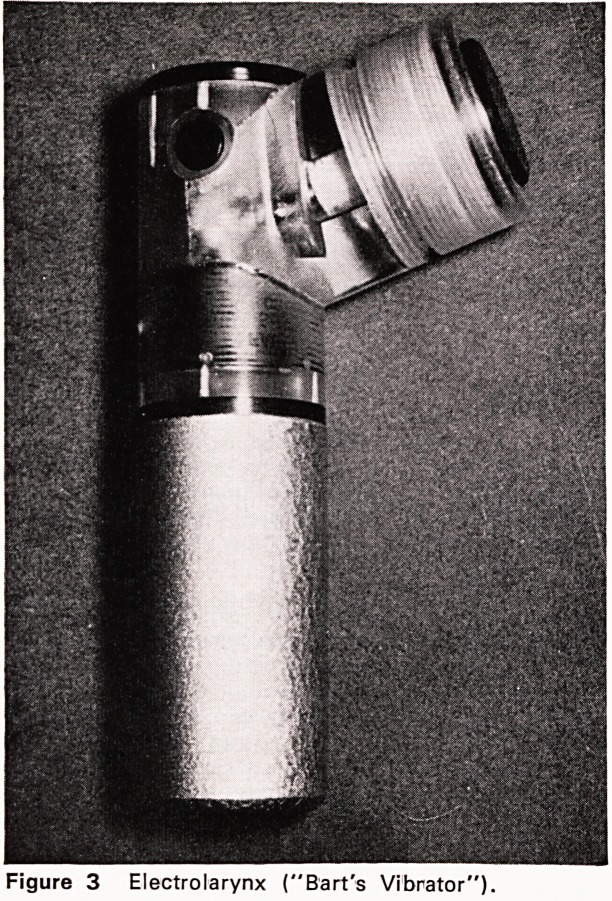


**Figure 4 f4:**
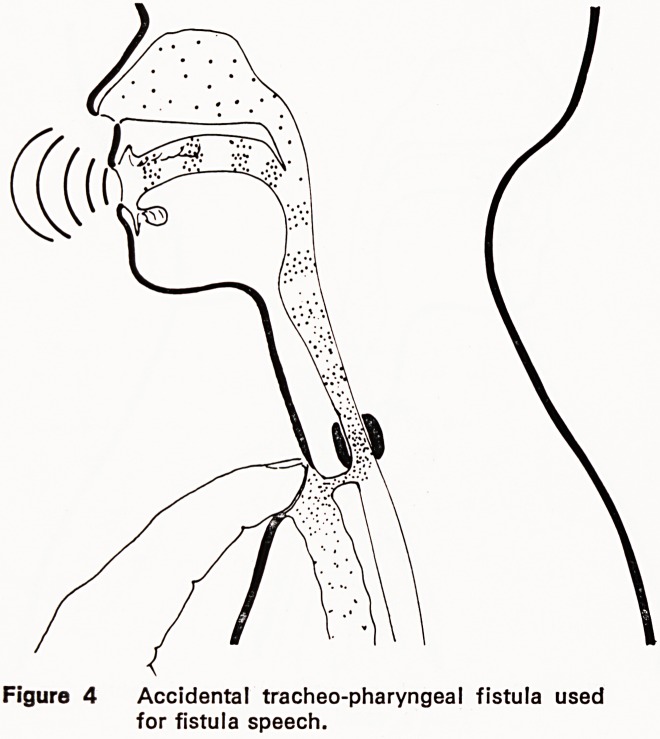


**Figure 5 f5:**
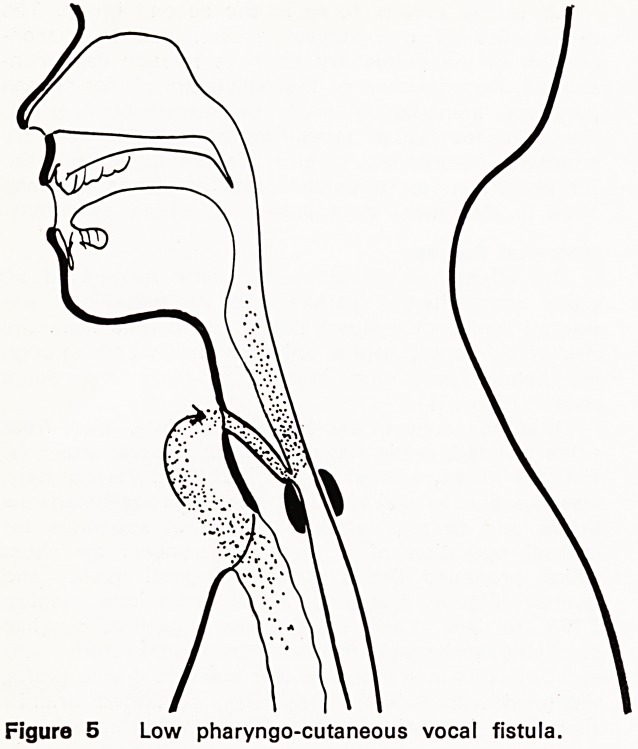


**Figure 6 f6:**
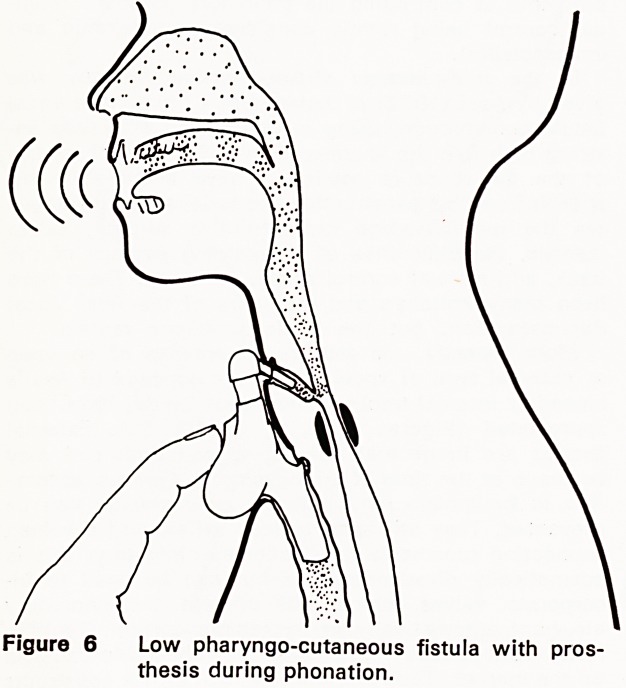


**Figure 7 f7:**
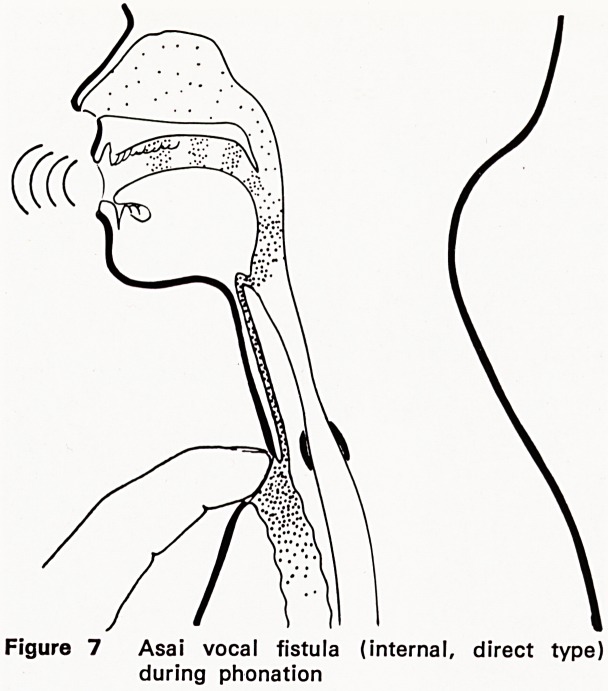


**Figure 8 f8:**
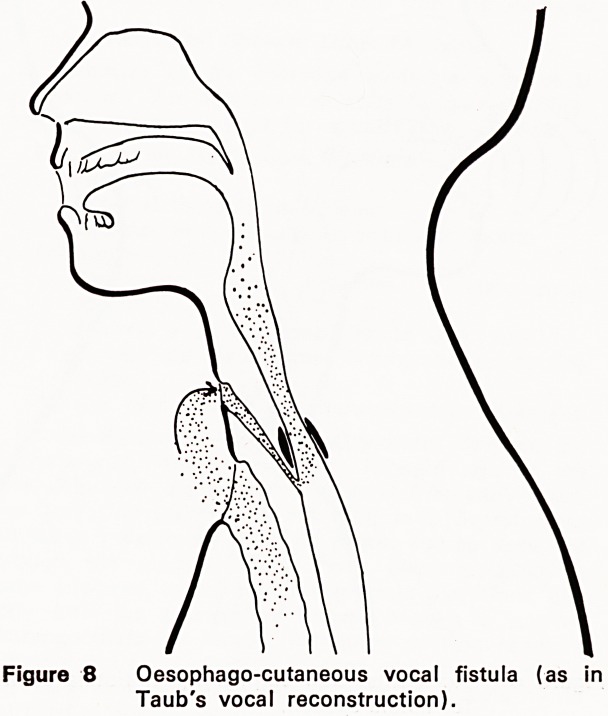


**Figure 9 Figure 10 f9:**
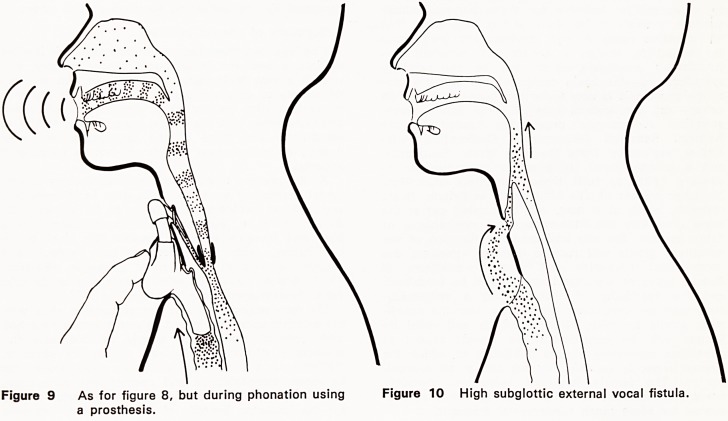


**Figure 11 f10:**